# Supplementary material for: Reconciling Mining with the Conservation of Cave Biodiversity: A Quantitative Baseline to Help Establish Conservation Priorities
Source: PLoS One. 2016 Dec 20;11(12):e0168348. doi: 10.1371/journal.pone.0168348 (PMC5173368; doi:10.1371/journal.pone.0168348)
Supplement: S1 Dataset — (ZIP) [file pone.0168348.s002.zip › Taxa/Serra Norte/SN_2013/N4WS/Lista N4WS-41.pdf]

## CAVIDADE N4WS-0041

| Classe | Ordem | Fam/Outros | Gên/Outros | Espécie | S-U | U-S | Zon. |
|---|---|---|---|---|---|---|---|
| Annelida | Oligochaeta | | | sp. | | 1 | |
| Arachnida | Amblypygi | Phrynidae | *Heterophrynus* | *longicornis* | 1 | | E |
| Arachnida | Araneae | Drymusidae | | jovem | X | | E |
| Arachnida | Araneae | Ochyroceratidae | *Speocera* | sp.1 | | X | E |
| Arachnida | Araneae | Pholcidae | *Mesabolivar* | *eberhard* | X | X | E |
| Arachnida | Araneae | Prodidomidae | | jovem | X | | P |
| Arachnida | Araneae | Theridiidae | | jovem | | X | P |
| Arachnida | Araneae | Theridiosomatidae | *Plato* | sp.1 | X | X | E/P |
| Arachnida | Pseudoscorpiones | Chernetidae | *Spelaeochernes* | sp.1 | X | X | E/P |
| Arachnida | Pseudoscorpiones | Chthoniidae | *Pseudochthonius* | sp.1 | | X | P |
| Chilopoda | Geophilomorpha | Ballophilidae | *Ityphilus* | sp.1 | | 1 | P |
| Diplopoda | Polydesmida | Fuhrmannodesmidae | | sp.1 | | X | E |
| Entognatha | Diplura | Campodeidae | | sp.1 | X | X | E |
| Insecta | Blattodea | Polyphagidae | | sp.1 | 1 | | P |
| Insecta | Coleoptera | Carabidae | | sp.5 | X | | E |
| Insecta | Coleoptera | Tenebrionidae | | jovem | X | | E |
| Insecta | Diptera | Ceratopogonidae | | jovem | X | X | E |
| Insecta | Diptera | Psychodidae | *Sciopemyia* | *sordellii* | X | | E |
| Insecta | Diptera | Tipulidae | Tipulinae | sp. | X | | E |
| Insecta | Heteroptera | Reduviidae | Emesinae | jovem | | 1 | E |
| Insecta | Heteroptera | Reduviidae | Reduviinae | jovem | 1 | 2 | E/P |
| Insecta | Homoptera | Cixiidae | | sp.5 | X | X | P |
| Insecta | Hymenoptera | Formicidae | *Apterostigma* | sp.1 | X | | P |
| Insecta | Hymenoptera | Formicidae | *Nylanderia* | sp.1 | X | | P |
| Insecta | Hymenoptera | Formicidae | *Nylanderia* | sp.3 | | X | E |
| Insecta | Hymenoptera | Formicidae | *Pheidole* | sp.2 | X | | E |
| Insecta | Isoptera | Termitidae | *Atlantitermes* | sp. | X | X | E |
| Insecta | Lepidoptera | Noctuoidea | | sp.2 | X | | E |
| Insecta | Orthoptera | Phalangopsidae | *Phalangopsis* | sp. | 8 | 8 | P |
| Insecta | Psocoptera | Psocomorpha | | jovem | | X | E |
| Malacostraca | Isopoda | Armadillidae | | sp.1 | X | | P |
| Amphibia | Anura | Strabomantidae | *Pristimantis* | cf. *fenestratus* | 2 | | E |
